# Upper and Lower Limb Muscle Architecture of a 104 Year-Old Cadaver

**DOI:** 10.1371/journal.pone.0162963

**Published:** 2016-12-29

**Authors:** Marissa Ruggiero, Daniel Cless, Benjamin Infantolino

**Affiliations:** Division of Science, Pennsylvania State University, Berks Campus, Reading, Pennsylvania, United State of America; Semmelweis Egyetem, HUNGARY

## Abstract

Muscle architecture is an important component to typical musculoskeletal models. Previous studies of human muscle architecture have focused on a single joint, two adjacent joints, or an entire limb. To date, no study has presented muscle architecture for the upper and lower limbs of a single cadaver. Additionally, muscle architectural parameters from elderly cadavers are lacking, making it difficult to accurately model elderly populations. Therefore, the purpose of this study was to present muscle architecture of the upper and lower limbs of a 104 year old female cadaver. The major muscles of the upper and lower limbs were removed and the musculotendon mass, tendon mass, musculotendon length, tendon length, pennation angle, optimal fascicle length, physiological cross-sectional area, and tendon cross-sectional area were determined for each muscle. Data from this complete cadaver are presented in table format. The data from this study can be used to construct a musculoskeletal model of a specific individual who was ambulatory, something which has not been possible to date. This should increase the accuracy of the model output as the model will be representing a specific individual, not a synthesis of measurements from multiple individuals. Additionally, an elderly individual can be modeled which will provide insight into muscle function as we age.

## Introduction

One of the first models of muscles that accounted for muscle architecture was developed by Niels Stensen in 1667 [1]. More recently, Gans and Bock [2] described how muscle architecture can be used to gain insight into a muscle’s function. Numerous authors have used this form and function relationship to describe the function of muscles [3–5]. Using this form/function relationship, many musculoskeletal models seek to estimate a muscle’s function based on its architecture [6]. Based on the estimated muscle function, these musculoskeletal models have been proposed to guide tendon transfer and other musculoskeletal surgeries [7]. Given the connection between a muscle’s form and its function the importance of obtaining muscle architectural measures is central in the understanding of muscle function and in the construction of musculoskeletal models for use in clinical areas such as rehabilitation and surgery.

Despite the importance of muscle architecture, the literature related to this area is limited. In many cases, a single study cannot provide all the parameters necessary for a musculoskeletal model and parameters from multiple studies must be used to construct a complete model [8]. The lower limb is better described in the literature than the upper with multiple studies reporting some muscle architectural parameters for most of the muscles in the lower limb [3, 9–13]. In the upper limb, studies focus on the neck [14], shoulder [15], elbow [16–19], and wrist [20–23]. No studies could be found that reported muscle architectural measures for the shoulder, elbow, and wrist musculature on a single cadaver. Finally, muscle architectural measurements from elderly cadavers are lacking, making it difficult to investigate the changes that occur with age and the effect these changes have on human movement.

In many of these studies at least one key component for the construction of a musculoskeletal model is missing. For example, in many of the previous studies resting tendon length was not reported yet resting tendon length is a critical parameter in musculoskeletal models [6]. Another limitation of previous studies is a lack of general information about the cadaver such as height, mass, age, gender, or cause of death. Height and mass are necessary to allow researchers to normalize the data to allow for comparisons between data sets or scaling of the presented data to live subjects. Gender and age are important for understanding how the data can be generalized to other populations. Finally, cause of death is important as some disease processes can lead to significant muscle wasting which can drastically change muscle mass measures while leaving other measures (such as tendon length) untouched.

Recently, researchers have begun focusing on developing highly detailed, complete, specific musculoskeletal data sets which are better able to be used to create a subject specific musculoskeletal model. These data sets are represented by the Twente Lower Extremity Model [11] and Twente Lower Extremity Model 2.0 [9]. Given this interest in creating highly detailed, complete, musculoskeletal data sets a data set is needed which combines both the upper and lower limbs. This highly detailed data set would allow for more subject specific whole body musculoskeletal models to be created.

The purpose of this study was to present the muscle architecture (musculotendon length, musculotendon mass, tendon length, tendon mass, pennation angle, and optimal fascicle length) for the major muscles of the upper and lower body on a single cadaver. This will be the first data set to date which presents muscle architecture for both the upper and lower limbs. Additionally this is the oldest specific cadaver in the literature by 13 years [3, 9–23].

## Materials and Methods

Procedures and measurements related to the cadaver were approved by The Pennsylvania State University Institutional Biosafety Committee. The need for informed consent was waived by the committee. The body donor was not from a vulnerable population and the donor or next of kin provided written informed consent that was freely given. The muscles of the right side of one embalmed female Caucasian cadaver (age at death: 104 years; mass: 73.4 kg; height: 1.69 m; cause of death: old age) were exposed from origin to insertion using blunt dissection. Once the entire musculotendon complex was exposed the musculotendon complex was removed from the body with care to remove all the muscle and tendon from the bones. All gross measures were made three times by the individual who removed the muscle to eliminate errors that could arise from reading a measure wrong once. In most cases all three measures were in agreement and in the rare case this was not the case, additional measures were taken by another individual to ensure an accurate measurement. Musculotendon complex mass (*M*_*mt*_) and tendon mass (*M*_*t*_) were recorded to the nearest 0.1 g immediately after removal. The musculotendon complex was placed against a standard rule and the musculotendon length (*L*_*mt*_) and tendon length (*L*_*t*_) were measured to the nearest 0.1 cm. Once the muscles were removed, palpable bony landmarks were measured to provide additional anthropmetric information about the cadaver and are presented in Table 1.

**Table 1:** Cadaver Anthropometric Measures.

Dimension	Measurement (cm)
**Femur length** *(greater trochanter to knee joint line)*	37.4
**Femoral condyle width** *(at widest point)*	7.6
**Tibia length** *(knee joint line to distal end of medial malleolus)*	32.4
**Tibial plateau width** *(at widest point)*	6.7
**Calcaneal width** *(at widest point)*	3.8
**Humerus length** *(shoulder joint line to elbow joint line)*	28.4
**Humerus condyle width** *(at widest point)*	6.3
**Radius length** *(elbow joint line to wrist joint line)*	22.1
**Hand length** *(wrist joint line to distal end of 3*^*rd*^ *finger)*	17.3
**Hand breath** *(width at 2*^*nd*^ *through 5*^*th*^ *MCP joints)*	7.5

Given the force-length properties of muscle [24] the length at which a muscle fascicle can produce maximal force was determined. Since muscle mass varies greatly across the human body [25] the number of fascicles sampled from each muscle was based upon its mass. Looking at previous research to determine the span of muscle masses it was determined that three fascicles would be removed for every 50 g of muscle mass. Muscles under 50 g would have three fascicles removed. These samples were taken from all parts of each muscle to ensure that the whole muscle was sampled equally. Once a fascicle was removed, its length (*L*_*f*_) was determined using a standard rule and recorded. Using a stereo dissection microscope and fine forceps, individual muscle fibers were removed from the fascicles. The muscle fiber was then mounted on a microscope slide and digital images of the muscle fiber at 400x magnification were taken. Custom written MATLAB code [26] (The Mathworks, Natick, MA) was used to measure average sarcomere length (*L*_*s*_). A minimum of 60 sarcomeres (averaged across all fascicle samples per muscle) was required to ensure adequate sarcomere sample size [15]. Optimal fascicle length was calculated using,

$$L_{fopt}=L_{f}\cdot \frac{L_{sopt}}{L_{s}}$$ (1)

Where *L*_*sopt*_ was 2.7 μm for human muscle fibers [27].

Pennation angle was measured using a clear plastic goniometer and recorded to the nearest degree when the muscle was first removed. However, since pennation angle varies with muscle fascicle length [28] pennation angle was reported at optimal fascicle length. To adjust the measured pennation angle to the pennation angle at optimal fascicle length a planimetric model of muscle was used and muscle thickness was assumed to remain constant for all fascicle lengths [5]. Based on this model, muscle thickness (*t*) was calculated using,

$$t=L_{f}\sin\theta$$ (2)

Where *θ* is the pennation angle measured directly after removal from the cadaver. Assuming that muscle thickness remains constant the pennation angle at optimum fascicle length (*θ*_*opt*_) was calculated using,

$$\theta_{opt}=\sin^{-1}\left(\frac{t}{L_{fopt}}\right)$$ (3)

Muscle force is directly proportional to PCSA which was calculated from Wickiewicz et al. [13],

$$PCSA=\frac{M_{m}\cdot \cos(\theta_{opt})}{\rho_{m}\cdot L_{fopt}}$$ (4)

Where *M*_*m*_ is the mass of the muscle, and *ρ*_*m*_ is the density of embalmed muscle [29] (1.112 g/cm^3^).

Tendons were approximated as having circular cross-sections which allowed for TCSA to be calculated using,

$$TCSA=\frac{M_{t}}{L_{t}\cdot \rho_{t}}$$ (5)

Where *ρ*_*t*_ is the density of tendon [30] (1.12 g/cm^3^).

The ratio of PCSA to TCSA was calculated to demonstrate how muscle force (approximated by PCSA) is related to the ability to resist/transmit the force to bone [31]. Additionally, the ratio of *L*_*t*_ to *L*_*fopt*_ was calculated to provide insight into the energy storage capacity of the muscles [32].

### Statistics

Descriptive statistics were used for the measured variables. Additionally, the ratio of PCSA/TCSA was calculated for each muscle. Common tendons (Achilles and Patellar) were included for each individual muscle since each muscle would produce a force on the common tendon. The coefficient of variation was calculated for the lower and upper limbs. To compare this variability against functionally similar groups of muscles the ratio of PCSA/TCSA was calculated for the quadriceps, hamstrings, plantarflexors, dorsiflexors, elbow flexors and extensors, and the finger flexors and extensors.

## Results

Table 2 presents the data from the upper limb while Table 3 presents the data from the lower limb. Unilateral absence of Palmaris longus was noted on the right side of the cadaver and therefore this muscle is not included in the analysis.

**Table 2:** Muscle Architecture of the Upper Limb.

Muscle Name	*M*_*mt*_ (g)	*M*_*t*_ (g)	*L*_*mt*_ (cm)	*L*_*t*_ (cm)	*θ*_*opt*_ (deg)	*L*_*f*_ (cm)	*L*_*s*_ (um)	# sarc	*L*_*fopt*_ (SD) (cm)	PCSA (cm^2^)	TCSA (cm^2^)	PCSA/TCSA	*L*_*t*_/*L*_*fopt*_
Abductor Pollicis Longus	6.7	1.0	20.3	7.5	9.6	3.3 (0.2)	2.60 (0.06)	78 (14)	3.4 (0.3)	1.5	0.1	12.4	2.2
Anconeus	3.0	0.0	7.5	0.0	0.0	1.5 (0.7)	1.94 (0.09)	69 (12)	2.1 (0.9)	1.3	0.0	-	-
Anterior Deltoid	32.6	0.5	18.6	5.4	29.8	6.8 (2.3)	1.88 (0.11)	80 (36)	9.7 (2.8)	2.6	0.1	31.2	0.6
Biceps Brachii Long Head	18.7	2.9	13.0	16.2	0.0	4.0 (0.7)	2.46 (0.03)	63 (14)	4.4 (0.7)	3.2	0.2	10.5	3.7
*Distal tendon*		1.3		7.9							0.1	-	-
Biceps Brachii Short Head	21.6	0.3	12.5	3.7	0.0	5.3 (1.0)	2.12 (0.13)	83 (25)	6.7 (1.1)	2.9	0.1	19.9	0.5
*Distal tendon*		0.5		6.4							0.1	-	-
Brachialis	50.5	0.0	18.7	0.0	0.0	5.8 (1.8)	3.52 (0.30)	74 (9)	4.5 (3.4)	10.1	0.0	-	-
Brachioradialis	19.1	1.4	29.9	8.9	0.0	6.1 (1.8)	2.21 (0.16)	77 (2)	7.3 (1.8)	2.2	0.1	15.5	1.2
Coracobrachialis	16.0	0.7	17.5	0.6	0.0	5.9 (0.6)	5.41 (0.05)	76 (32)	8.8 (1.1)	1.6	1.0	1.6	0.1
Extensor Carpi Radialis Brevis	12.1	1.1	24.1	10.2	11.5	4.9 (0.4)	2.09 (0.06)	87 (10)	6.3 (0.5)	1.5	0.1	15.9	1.6
Extensor Carpi Radialis Longus	13.7	1.9	28.1	18.1	0.0	7.6 (1.5)	2.27 (0.04)	86 (4)	9.0 (1.9)	1.2	0.1	12.6	2.0
Extensor Carpi Ulnaris	11.9	0.6	25.6	5.4	0.0	2.9 (0.3)	3.43 (0.05)	75 (11)	2.3 (0.3)	4.4	0.1	44.5	2.3
Extensor Digiti Minimi	2.3	0.3	19.1	9.5	0.0	3.4 (0.8)	2.93 (0.08)	90 (10)	3.1 (0.7)	0.6	0.0	20.6	3.1
Extensor Digitorum 2	5.5	3.8	34.9	21.6	0.0	4.4 (0.8)	2.96 (0.17)	85 (4)	4.0 (0.6)	0.4	0.2	2.4	5.4
*Proximal tendon*		1.1		1.3							0.8	-	-
Extensor Digitorum 3	5.6	2.1	38.1	27.2	0.0	4.9 (0.3)	2.82 (0.14)	98 (9)	4.7 (0.5)	0.7	0.1	9.7	5.8
Extensor Digitorum 4	5.4	1.6	33.2	21.0	0.0	4.9 (0.4)	2.85 (0.12)	92 (9)	4.7 (0.6)	0.7	0.1	10.7	4.5
Extensor Digitorum 5	5.4	1.2	31.8	14.1	0.0	3.7 (1.6)	3.30 (0.07)	69 (13)	3.0 (1.3)	1.3	0.1	16.6	4.7
Extensor Indicis	3.3	1.1	26.0	15.9	0.0	4.6 (0.7)	2.63 (0.20)	70 (9)	4.7 (0.7)	0.4	0.1	6.8	3.4
Extensor Pollicis Brevis	24.5	0.4	21.1	11.6	0.0	2.5 (0.3)	2.60 (0.20)	80 (9)	2.7 (0.5)	0.7	0.0	21.6	4.3
Extensor Pollicis Longus	4.1	0.8	26.7	11.6	0.0	3.3 (1.8)	2.75 (0.15)	82 (3)	3.2 (1.6)	0.9	0.1	15.0	3.6
Flexor Carpi Radialis	14.2	1.3	27.3	9.5	0.0	4.1 (0.3)	1.65 (0.17)	99 (26)	6.8 (1.2)	1.7	0.1	14.0	1.4
Flexor Carpi Ulnaris	17.1	0.5	24.6	1.9	3.1	4.3 (1.3)	1.69 (0.09)	85 (18)	7.0 (2.4)	2.1	0.2	9.1	0.3
Flexor Digitorum Profundus 2	10.3	1.8	32.4	16.8	4.6	6.2 (1.0)	2.46 (0.11)	80 (17)	6.8 (1.1)	1.1	0.1	11.7	2.5
Flexor Digitorum Profundus 3	15.4	2.3	36.4	14.5	8.8	7.3 (0.3)	2.39 (0.32)	92 (36)	8.2 (1.0)	1.4	0.1	10.0	1.8
Flexor Digitorum Profundus 4	12.2	1.3	37.8	12.7	4.8	7.5 (0.9)	2.58 (0.07)	115 (5)	7.8 (0.8)	1.3	0.1	13.7	1.6
Flexor Digitorum Profundus 5	9.8	1.2	32.7	14.6	5.6	6.3 (0.2)	2.55 (0.32)	84 (52)	6.7 (0.7)	1.1	0.1	15.7	2.2
Flexor Digitorum Superficialis 2	10.7	1.1	35.9	14.9	7.4	4.7 (0.5)	1.86 (0.38)	93 (47)	6.9 (0.9)	1.2	0.1	18.8	2.2
Flexor Digitorum Superficialis 3	30.3	1.8	38.6	13.3	7.0	7.9 (0.0)	1.72 (0.07)	91 (22)	12.5 (0.5)	2.0	0.1	16.9	1.1
Flexor Digitorum Superficialis 4	9.6	1.2	34.1	14.9	3.8	4.9 (2.7)	1.69 (0.06)	89 (17)	7.7 (1.1)	1.0	0.1	13.6	1.9
Flexor Digitorum Superficialis 5	3.3	0.5	31.9	10.5	3.0	5.4 (0.4)	1.62 (0.08)	93 (22)	9.0 (0.6)	0.3	0.0	6.6	1.2
*Proximal Tendon*		0.1		5.7							0.0	-	-
Flexor Pollicis Longus	8.3	1.2	27.6	11.8	10.1	4.3 (0.5)	2.47 (0.24)	95 (32)	4.7 (0.3)	1.3	0.1	14.7	2.5
Infraspiantus	53.1	0.3	16.5	1.6	17.5	4.9 (0.9)	2.62 (0.15)	92 (13)	4.2 (0.8)	10.8	0.2	64.0	0.4
Latissimus Dorsi	163.3	0.0	38.7	0.0	21.8	5.9 (2.2)	2.42 (0.31)	81 (24)	6.7 (2.7)	20.3	0.0	-	-
Middle Deltoid	77.5	0.0	16.8	0.0	0.0	6.4 (1.7)	1.87 (0.13)	88 (22)	9.2 (2.5)	7.6	0.0	-	-
Pectoralis Major	105.5	0.0	18.2	0.0	39.2	7.9 (1.6)	2.53 (0.71)	81 (15)	9.1 (3.4)	8.1	0.0	-	-
Pectoralis Minor	15.4	0.8	15.4	1.0	11.8	7.1 (0.6)	2.16 (0.14)	62 (14)	9.0 (1.3)	1.4	0.7	2.0	0.1
Posterior Deltoid	30.3	1.9	19.7	6.2	10.0	5.0 (0.3)	1.61 (0.03)	93 (22)	8.4 (0.5)	3.0	0.3	10.9	0.7
Pronator Quadratus	3.9	0.0	3.8	0.0	0.0	1.8 (0.5)	2.14 (0.12)	117 (27)	2.3 (0.7)	1.5	0.0	-	-
Pronator Teres	3.0	0.1	11.9	1.0	0.0	2.6 (0.6)	2.17 (0.25)	108 (28)	3.4 (1.2)	0.8	0.1	8.2	0.3
Rhomboid Major	28.3	0.0	12.7	0.0	0.0	5.1 (3.1)	2.31 (0.17)	120 (12)	5.8 (3.1)	4.4	0.0	-	-
Rhomboid Minor	5.0	0.0	8.9	0.0	0.0	3.1 (0.9)	7.02 (0.09)	78 (25)	3.6 (1.2)	1.2	0.0	-	-
Serratus Anterior	79.8	0.0	27.0	0.0	16.8	3.5 (0.7)	3.06 (0.30)	82 (11)	3.1 (0.6.)	22.2	0.0	-	-
Subscapularis	64.5	0.0	12.1	0.0	15.5	3.0 (0.7)	2.03 (0.23)	104 (23)	3.8 (0.8)	14.7	0.0	-	-
Supinator	9.8	0.0	8.9	0.0	0.0	2.2 (0.2)	2.79 (0.43)	75 (23)	2.3 (0.5)	3.8	0.0	-	-
Supraspinatus	28.5	0.0	10.8	0.0	0.0	6.1 (0.4)	3.29 (0.10)	81 (12)	5.0 (0.4)	5.1	0.0	-	-
Teres Major	35.3	0.0	11.0	0.0	0.0	2.8 (0.6)	2.12 (0.26)	86 (23)	3.6 (0.9)	8.8	0.0	-	-
Teres Minor	10.4	0.4	11.6	1.3	0.0	3.9 (1.4)	2.83 (0.46)	82 (16)	3.7 (1.2)	2.4	0.3	8.6	0.3
Trapezius	97.2	0.0	33.7	0.0	43.5	4.9 (1.8)	2.56 (0.38)	96 (22)	5.0 (1.3)	12.7	0.0	-	-
Triceps Brachii Lateral Head	44.0	0.0	24.8	0.0	0.0	3.7 (0.7)	2.18 (0.24)	109 (36)	4.7 (1.3)	8.4	0.0	-	-
Triceps Brachii Long Head	53.8	1.6	25.4	3.2	0.0	3.8 (0.5)	2.01 (0.13)	99 (24)	5.2 (0.7)	9.0	0.4	20.1	0.6
Triceps Brachii Medial Head	42.7	0.0	20.3	0.0	0.0	2.5 (1.0)	2.10 (0.20)	59 (5)	3.2 (1.5)	12.0	0.0	-	-

**Table 3:** Muscle Architecture of the Lower Limb.

Muscle Name	*M*_*mt*_ (g)	*M*_*t*_ (g)	*L*_*mt*_ (cm)	*L*_*t*_ (cm)	*θ*_*opt*_ (deg)	*L*_*f*_ (SD) (cm)	*L*_*s*_ (SD) (um)	# sarc (SD)	*L*_*fopt*_ (SD) (cm)	PCSA (cm^2^)	TCSA (cm^2^)	PCSA/TCSA	*L*_*t*_/*L*_*fopt*_
Adductor Brevis	109.4	0.0	18.4	0.0	0.0	8.3 (0.5)	3.00 (0.28)	82 (9)	7.5 (0.6)	13.1	0.0	-	-
Adductor Longus	91.7	1.0	32.1	2.5	0.0	7.0 (1.4)	3.63 (0.19)	67 (13)	5.3 (1.2)	15.4	0.4	43.8	0.5
Adductor Magnus	185.6	0.0	22.1	0.0	0.0	11.3 (2.0)	2.08 (0.19)	103 (29)	14.9 (3.2)	11.2	0.0	-	-
Biceps Femoris Long Head	106.4	2.4	45.4	13.3	6.1	7.8 (1.4)	2.06 (0.27)	86 (13)	10.2 (1.6)	8.8	0.2	17.1	2.1
*Proximal tendon*		3.3		8.3							0.4		
Biceps Femoris Short Head	58.6	1.4	32.7	2.9	24.5	15.2 (2.3)	2.99 (0.16)	84 (12)	13.7 (2.0)	3.4	0.4	7.9	0.2
Extensor Digitorum Longus	36.6	4.6	45.7	15.4	16.3	6.6 (0.5)	3.15 (0.18)	78 (19)	5.7 (0.7)	4.8	0.3	18.0	2.7
Extensor Hallucis Longus	17.9	1.0	37.0	13.2	12.2	7.8 (0.8)	3.30 (0.08)	94 (21)	6.4 (0.8)	2.3	0.1	34.3	2.1
Flexor Digitorum Longus	14.9	2.9	43.7	19.7	12.0	4.6 (1.6)	2.32 (0.15)	114 (18)	5.3 (2.1)	2.0	0.1	14.9	3.7
Flexor Hallucis Longus	35.9	2.5	34.0	21.4	15.9	3.5 (0.4)	2.41 (0.06)	89 (13)	4.0 (0.6)	7.2	0.1	69.3	5.4
Gastrocnemius Lateral Head	96.3	1.2	37.9	11.6	6.7	6.5 (1.1)	1.81 (0.11)	85 (15)	9.6 1.2)	8.8	0.1	13.2	1.8
*Achilles Tendon*		3.8		5.9							0.6		
Gastrocnecmius Medial Head	69.4	3.3	34.6	15.1	14.4	6.6 (1.0)	2.04 (0.40)	66 (17)	9.0 (2.3)	6.4	0.2	8.3	2.3
*Achilles Tendon*		3.8		5.9							0.6		
Gemellus Inferior	8.2	0.0	7.3	0.0	0.0	2.2 (0.3)	2.02 (0.36)	66 (3)	3.0 (0.4)	2.5	0.0	-	-
Gemellus Superior	6.8	0.7	10.2	1.6	0.0	2.8 (0.2)	1.89 (0.15)	98 (33)	3.9 (0.4)	1.4	0.4	3.6	0.4
Gluteus Maximus	522.0	0.0	26.0	0.0	0.0	10.9 (2.8)	2.38 (0.31)	81 (19)	12.6 (3.5)	37.3	0.0	-	-
Gluteus Medius	189.1	0.0	19.7	0.0	53.0	6.4 (0.8)	3.32 (0.62)	82 (18)	6.1 (0.8)	16.8	0.0	-	-
Gluteus Minimus	49.6	0.1	12.1	1.3	87.1	3.9 (0.7)	3.54 (0.02)	73 (15)	2.9 (0.5)	2.2	0.1	31.4	0.4
Gracilis	59.5	2.1	51.8	13.0	0.0	23.8 (7.3)	2.77 (0.17)	93 (8)	19.6 (5.9)	2.6	0.1	18.3	0.7
Iliacus	43.3	0.0	22.1	0.0	0.0	10.9 (0.5)	3.29 (0.34)	88 (15)	9.0 (1.4)	4.3	0.0	-	-
Obturator Externus	38.5	0.4	12.7	1.9	26.8	6.3 (0.3)	2.90 (0.26)	102 (12)	5.9 (0.9)	5.2	0.2	27.6	0.3
Obturator Internus	38.0	1.9	16.2	6.4	15.5	4.0 (0.7)	2.01 (0.12)	80 (30)	5.4 (0.8)	5.8	0.3	21.7	1.2
Pectineus	20.4	0.0	11.7	0.0	0.0	10.3 (0.7)	3.27 (0.16)	79 (3)	8.5 (0.4)	2.2	0.0	-	-
Peroneus Brevis	14.2	0.9	36.8	8.3	0.0	3.5 (1.3)	2.86 (0.11)	87 (4)	3.3 (1.2)	3.5	0.1	25.9	4.5
*Proximal tendon*		0.3		6.7							0.0		
Peroneus Longus	32.1	3.2	39.1	18.4	25.1	3.4 (1.3)	2.82 (0.10)	108 (3)	3.3 (1.2)	7.6	0.2	49.2	5.6
Piriformis	17.4	0.0	10.3	0.0	7.2	5.0 (0.4)	2.15 (0.10)	96 (26)	6.3 (0.7)	2.5	0.0	-	-
Plantaris	7.5	0.5	43.0	32.7	6.1	4.4 (0.4)	2.72 (0.15)	76 (36)	4.4 (0.5)	1.4	0.0	104.2	7.4
Popliteus	11.8	1.8	13.0	2.9	0.0	3.3 (0.5)	3.02 (0.16)	92 (8)	3.0 (0.6)	3.0	0.6	5.2	1.0
Psoas Major	77.4	1.3	31.4	4.9	18.5	11.5 (0.4)	2.96 (0.34)	85 (12)	10.6 (0.9)	6.1	0.0	25.8	0.5
Psoas Minor	55.2	1.6	22.4	10.8	0.0	11.8 (0.9)	3.13 (0.29)	80 (12)	10.3 (1.4)	4.7	0.1	35.4	1.0
Quadratus Femoris	25.6	0.0	6.7	0.0	0.0	3.3 (0.6)	1.97 (0.30)	101 (7)	4.6 (0.6)	5.0	0.0	-	-
Rectus Femoris	88.7	2.3	40.0	6.4	9.5	6.9 (0.6)	2.58 (0.33)	81 (12)	7.3 (0.9)	10.5	0.3	10.8	1.4
*Patellar tendon*		2.9		4.0							0.7		
Sartorius	81.0	0.9	57.0	3.0	0.0	41.2 (2.8)	2.92 (0.22)	84 12)	38.2 (2.3)	1.9	0.3	5.8	0.2
*Proximal tendon*		0.2		3.2							0.1		
Semimembranosus	119.9	2.6	41.1	4.4	32.2	5.3 (1.6)	2.44 (0.31)	85 (12)	6.0 (1.9)	13.7	0.5	11.9	2.9
*Proximal tendon*		9.0		12.9							0.6		
Semitendanosus	90.0	2.6	48.3	18.3	0.0	8.4 (0.7)	2.52 (0.19)	104 (12)	9.0 (0.4)	8.7	0.1	68.7	2.0
Soleus	230.8	0.0	27.5	0.0	22.9	4.5 (0.4)	2.11 (0.13)	72 (17)	5.8 (0.6)	33.0	0.0	57.0	1.0
*Achilles Tendon*		3.8		5.9							0.6		
Tensor Fasciae Latae	25.4	0.0	15.2	0.0	0.0	12.5 (0.2)	2.59 (0.70)	102 (4)	13.8 (4.3)	1.7	0.0	-	-
Tibialis Anterior	62.7	5.2	36.2	17.8	3.9	8.3 (1.7)	2.10 (0.20)	82 (16)	10.6 (2.1)	4.9	0.3	18.8	1.7
Tibialis Posterior	41.9	2.9	36.4	10.0	9.7	3.3 (0.2)	2.40 (0.29)	86 (23)	3.7 (0.3)	9.3	0.3	36.1	2.7
Vastus Intermedius	163.9	0.0	35.2	0.0	9.4	6.7 (0.9)	2.13 (0.18)	105 (20)	8.5 (1.4)	17.1	0.0	26.2	0.5
*Patellar tendon*		2.9		4.0							0.7		
Vastus Lateralis	149.3	1.6	36.2	4.1	8.2	6.6 (1.0)	2.21 (0.27)	103 (19)	8.0 (1.0)	16.4	0.3	16.5	1.0
*Patellar tendon*		2.9		4.0							0.7		
Vastus Medialis	135.7	0.0	28.9	0.0	24.5	7.8 (0.8)	2.25 (0.20)	105 (18)	9.4 (1.0)	11.8	0.0	18.1	0.4
*Patellar tendon*		2.9		4.0							0.7		

The total PCSA of major muscle groups were compared. For the lower limb, the total PCSA of the quadriceps was larger than that of the hamstrings (55.9 cm^3^ vs. 34.7 cm^3^) while the PCSA of the plantarflexors was greater than that of the dorsiflexors (45.0 cm^3^ vs. 12.0 cm^3^). In the upper limb, the PCSA of the elbow extensors was larger than that of the flexors (20.1 cm^3^ vs. 9.8 cm^3^) while the PCSA of the finger flexors was larger than that of the extensors (9.5 cm^3^ vs. 3.0 cm^3^).

Elliott and Crawford [31] reported that the PCSA and TCSA of muscles are not always strictly correlated. Muscles were grouped according to their gross function (knee flexion, hip extension, etc.) to determine if as a group the coefficient of variation was relatively low and therefore for functional groups of muscles the PCSA and TCSA values would be strictly correlated. Table 4 presents the coefficient of variation data.

**Table 4:** Coefficient of Variation of PCSA/TCSA for Lower and Upper Limbs and Functionally Similar Groups.

Group	Coefficient of Variation (%)
Lower Limb	80.5
Upper Limb	76.1
Quadriceps	35.7
Hamstrings	107.6
Plantarflexors	82.3
Dorsiflexors	38.7
Elbow Flexors	66.0
Elbow Extensors	NA
Finger Flexors	29.4
Finger Extensors	59.1

Note that the elbow extensors do not have a coefficient of variation since only one of the Triceps brachii was found to have a tendon.

The ratio of *L*_*t*_/*Lf*_*opt*_ was calculated for each muscle and again common tendons were included for each individual muscle. The average ratio was calculated for the lower and upper limb as well as for the quadriceps, hamstrings, plantarflexors, dorsiflexors, elbow flexors and extensors, and the finger flexors and extensors. Table 5 presents the mean and standard deviation of the *L*_*t*_/*Lf*_*opt*_ ratio data.

**Table 5:** Mean and Standard Deviation of *L*_*t*_/*Lf*_*opt*_ Ratio for Different Groups of Muscles.

Group	Mean ± SD
Lower Limb	1.4 ± 1.8
Upper Limb	2.1 ± 1.6
Quadriceps	0.8 ± 0.5
Hamstrings	1.8 ± 1.1
Plantarflexors	4.2 ± 1.9
Dorsiflexors	2.1 ± 0.5
Elbow Flexors	1.4 ± 1.6
Elbow Extensors	0.6
Finger Flexors	1.8 ± 0.5
Finger Extensors	4.5 ± 1.1

Note the elbow extensors does not have a standard deviation since only one head of the Triceps brachii muscle had a significant tendon attached.

## Discussion

The musculoskeletal architecture of major muscles for the upper and lower right limb of a 104 year old female cadaver was presented. This is the first data set that combines an upper and lower limb from one single cadaver. In many cases, data (whole or partial) on muscles presented in the current study have been presented for other cadavers elsewhere. S1, S2, and S3 tables in the Supplemental Material present all the data from the major cadaver studies undertaken since 1975. S4 provides notes pertaining to the other three Supplemental files. Where possible, normalized data is presented for further comparisons to be drawn. Unfortunately, in many cases no data is presented in previous studies which would allow for normalization. This greatly reduces the ability to generalize the data to other populations.

Comparing PCSA values from this study to previous studies (Supplemental Tables 1 and 2) it was noted that in some cases the PCSA from this study was the largest, sometimes the smallest, and other times in the middle. Considering the cadaver in the current study is the oldest out of the studies represented in the supplemental tables this suggests that one cannot assume that all PCSA values can simply be scaled up or down to reflect changes in muscle function with age. Looking at normalized parameters across previous studies (supplemental Table 3) when muscle mass is normalized by body mass and muscle length, tendon length and optimal fascicle length are normalized by body height no discernable pattern can be found either. In some muscles the tallest cadaver has the largest normalized length values while in other muscles the tallest cadaver will have the smallest normalized values. This further underscores the error in using simple scaling parameters for all muscles when scaling a model to a subject’s height or mass.

The ratio of PCSA/TCSA was shown to have a large amount of variability across different muscles in the current study which is consistent with the finding of Elliott and Crawford [31]. While this may be explained by different muscles having different functional roles in movement, a similar finding has been reported previously in a study focused on a single muscle [33]. Cutts et al. [21] and Langenderfer et al. [15] both presented PCSA and TCSA values for muscles in the arm which allows for comparisons among the two previous studies and the current one. Cutts et al. [21] reported PCSA and TCSA for muscles of the wrist and fingers. There was a significant difference (p < 0.05) between the PCSA/TCSA ratios between the study by Cutts et al. and the current study. Looking closer at the data reveled a significant difference (p < 0.05) in PCSA values yet no difference (p > 0.05) in TCSA values. Langenderfer et al. [15] reported PCSA and TCSA values for muscles of the upper limb. Only eight muscles could be compared due to missing TCSA values in one study or the other. However, there was no statistically significant difference (p > 0.05) between PCSA, TCSA, or PCSA/TCSA values which is in contrast to the comparison of the data from Cutts et al. The data from Cutts et al. came from a man who was “about 20 years” old and who’s arm had to be amputated midway along the humerus due to a train accident. The two upper extremities from the study by Langenderfer et al. came from a 28 year old 65.9 kg cadaver and a 91 year old 63.6 kg cadaver. No cause of death was given for either cadaver. The cadavers presented in Langenderfer et al. were female which may explain why the values did not differ between their study and the current study whereas the values from the male subject in Cutts et al. did. The cadaver in the current study had a larger mass than either of the cadavers from Langenderfer et al. Whole body mass was not given in Cutts et al. so this comparison cannot be continued. It is possible that age was a factor between the present study and Cutts et al., however, one cadaver from Langenderfer et al. was a similar age to that of the individual in Cutts et al. which tends to indicate that age may not have been a factor. Unfortunately, no muscles overlapped between Langenderfer et al. and Cutts et al. so comparisons between the two studies cannot be made. Ultimately, not enough information exists between the three studies to provide a clear understanding of how PCSA, TCSA, and PCSA/TCSA ratio are influenced by age, gender, or cause of death.

The cause of death is an important factor in muscle architecture studies. In many cases, a disease that causes death will also have a negative effect on muscle mass and tendon properties due to decreased use of muscles. This change can skew the architectural data presented and if the primary cause of death is not reported it can be difficult to know how the data would compare to a living individual. In this study the reported cause of death was old age which, if nothing else, seems to indicate that the individual was relatively healthy (not under the care of a physician for a serious illness). Obviously this is purely conjecture and more detailed medical records would be extremely useful but it gives some insight into the relative quality of the muscle and tendons of the cadaver.

This study is the first of its kind in that it reported muscle architectural measures for the major muscles of the upper and lower limbs. Additionally, the cadaver in this study is the oldest reported individual female cadaver by 13 years for the upper limb [15] and 41 years for the lower limb [10]. This type of data should aid in producing whole body musculoskeletal models that are more representative of subjects. Variability in muscle architecture parameters has been demonstrated in other studies [12, 33]. Unfortunately, since only one cadaver was used in the current study the variability of upper and lower limb muscle architectural parameters could not be assessed.

Some limitations existed in this study. One limitation was the assumption of sarcomere length homogeneity. Infantolino et al. [26] showed that sarcomere length inhomogeneity existed for the First Dorsal Interosseous muscle and that large sample sizes of sarcomeres are necessary to accurately estimate optimal fascicle length. This is in contrast to Langenderfer et al. [15] who estimated that 40–60 sarcomeres are necessary to accurately estimate optimal fascicle length using a bootstrap simulation. Given the number of muscles in this study the recommendation of Langenderfer et al. was followed realizing that this would introduce some error into our optimal fascicle length calculations. Shrinkage of muscle tissue was not an issue since the muscles was fixed while still attached to the bone [34] and any shrinkage would have been corrected for when optimal fascicle length was calculated. A second limitation was the use of only one cadaver in this study. While this is indeed a limitation and is not representative of the whole population; as discussed above, much of the previous cadaveric work is lacking critical information and therefore it has not been possible to construct a musculoskeletal model of both limbs that was based on a single individual who lived.

The results of this study present the first dataset to include both the upper and lower limb musculature of a cadaver. This data provides the necessary muscle architectural information needed to construct a whole body musculoskeletal model of a single individual who was ambulatory. Additionally, this data increases the body of knowledge of muscle architectural parameters in an elderly population. This will allow for the creation of more realistic models to investigate the effects of aging on human movement.

## Supporting Information

S1 Table Upper Limb Muscle Comparisons (XLSX)

S2 Table Lower Limb Muscle Comparisons (XLSX)

S3 Table Normalized Muscle Comparisons (XLSX)

S4 Table Supplemental Data Notes (DOCX)
