# Supplementary material for: Upper and Lower Limb Muscle Architecture of a 104 Year-Old Cadaver
Source: PLoS One. 2016 Dec 29;11(12):e0162963. doi: 10.1371/journal.pone.0162963 (PMC5199092; doi:10.1371/journal.pone.0162963)
Supplement: S4 Table — (DOCX) [file pone.0162963.s004.docx]

Supplemental Material Notes

Table legend

* - denotes reported resting length, not optimal fascicle length

† - denotes calculated value based on data and equations provided in original manuscript text

For certain muscles (e.g. Gluteus maximus, Latissmus dorsi) some authors have chosen to split the muscles into multiple sections. The maximum number of splits is presented, however for muscles with less splits the data is listed on the first line of the muscle.

The Pectoralis Major muscle in this study did not clearly separate into a clavicular and sternal head, therefore the muscle was treated as one muscle.

Standard deviations when provided are presented in parentheses.

Muscles that were combined are noted in the table.

Muscles with proximal and distal tendons are presented proximal/distal, except for Gastrocnemius and Soleus which is presented as tendon/Achilles tendon and quadriceps which is presented as tendon/Patellar tendon.
